# Modulation of Human Immune Cells by Propyl-Propane Thiosulfonate (PTSO) Inhibits Colorectal Tumor Progression in a Humanized Mouse Model

**DOI:** 10.3390/nu17182993

**Published:** 2025-09-18

**Authors:** María Jesús Rodríguez-Sojo, Luckman Gbati, Jose Alberto Molina-Tijeras, Ailec Ho-Plágaro, Teresa Vezza, Laura López-Escánez, Carmen Griñán-Lisón, Juan Antonio Marchal, Alberto Baños, María José Rodríguez-Sánchez, Jorge García-García, Antonio Jesús Ruiz-Malagón, Julio Gálvez, María Elena Rodríguez-Cabezas, Alba Rodríguez-Nogales

**Affiliations:** 1 Department of Pharmacology, Center for Biomedical Research (CIBM), University of Granada, 18071 Granada, Spain; mariajesus.rodriguez.sojo@gmail.com (M.J.R.-S.); cogitoterra@gmail.com (L.G.); jalbertomolinatijeras@gmail.com (J.A.M.-T.); ailec_hp@hotmail.com (A.H.-P.); e.lle197@go.ugr.es (L.L.-E.); mjrs2188@gmail.com (M.J.R.-S.); jgalvez@ugr.es (J.G.); merodri@ugr.es (M.E.R.-C.); albarn@ugr.es (A.R.-N.); 2 Instituto de Investigación Biosanitaria ibs.GRANADA, 18012 Granada, Spain; teresavezza@hotmail.it (T.V.); glcarmex@gmail.com (C.G.-L.); jmarchal@ugr.es (J.A.M.); 3 Digestive System Service, Virgen de las Nieves University Hospital, 18014 Granada, Spain; 4 Department of Biochemistry and Molecular Biology II, Faculty of Pharmacy, Campus de Cartuja s/n, University of Granada, 18016 Granada, Spain; 5 GENYO, Centre for Genomics and Oncological Research, Pfizer/University of Granada/Andalusian Regional Government, 18016 Granada, Spain; 6 Excellence Research Unit “Modeling Nature” (MNat), Centre for Biomedical Research (CIBM), University of Granada, 18071 Granada, Spain; 7 Biopathology and Regenerative Medicine Institute (IBIMER), Center for Biomedical Research (CIBM), University of Granada, 18016 Granada, Spain; 8 Department of Human Anatomy and Embryology, Faculty of Medicine, University of Granada, 18016 Granada, Spain; 9 DMC Research Center, 18620 Granada, Spain; abarjona@domca.com; 10 Servicio Microbiología, Hospital Universitario Clínico San Cecilio, 18100 Granada, Spain; 11 Centro de Investigación Biomédica en Red-Enfermedades Hepáticas y Digestivas (CIBER-EHD), 28029 Madrid, Spain

**Keywords:** immune checkpoint modulation, phytochemical, 3D CRC spheroid models, myeloid-derived suppressor cells, cancer adjuvant therapy, cytotoxic T lymphocyte, tumor xenograft mouse model

## Abstract

**Background/Objectives**: Colorectal cancer (CRC) remains a major global health challenge and current therapies are not always effective. In addition, certain immune cell populations, such as myeloid-derived suppressor cells (MDSCs), pose a significant barrier to immune-based treatments. Some phytochemicals, particularly compounds derived from *Allium* spp. like Propyl-Propane Thiosulfonate (PTSO), have shown strong immunomodulatory potential in digestive disorders. This study aims to investigate the capacity of PTSO to modulate immune responses and affect tumor progression in CRC models, in vitro and in vivo, with a focus on the immune cell populations that comprise the tumor microenvironment. **Methods**: Human peripheral blood mononuclear cells (hPBMCs) were incubated with PTSO (25 μM for 48 h) and characterized by flow cytometry. These cells (1 × 10^6^) were then injected into NOD scid gamma (NSG) immunodeficient mice, which were simultaneously induced to develop a subcutaneous tumor by injection of HCT116 enriched cancer stem cells (CSCs) colonospheres (60,000 cells/mouse). **Results**: PTSO reduced MDSC populations, specifically, it significantly reduced monocytic (M-MDSCs, Control: 7.27 ± 0.53% vs. PTSO: 4.70 ± 2.39%; *p* = 0.0458) and polymorphonuclear (PMN-MDSCs, Control: 5.28 ± 0.99% vs. PTSO: 3.41 ± 1.58%; *p* = 0.0385) MDSCs. In parallel, PTSO increased T cell subpopulations, particularly interferon gamma (IFNG)-producing cytotoxic CD8^+^ T cells (Control: 9.52 ± 2.06% vs. PTSO: 15.04 ± 5.01%; *p* = 0.0685). In the humanized tumor xenograft mouse, the administration of PTSO-pretreated hPBMCs led to a significant reduction in tumor size (Control: 1.43 ± 0.82 cm^3^ vs. PTSO: 0.44 ± 0.35 cm^3^; *p* = 0.0068), accompanied by increased infiltration of CD4^+^ T lymphocytes and Natural Killer (NK) cells and downregulation of immunosuppressive genes. These effects resulted in a reduction in cancer cell proliferation and invasiveness. **Conclusions**: The dual effect of PTSO on immune cell populations, reducing immunosuppressive myeloid cells and enhancing effector T lymphocyte and NK cell responses, resulted in an anti-tumor effect, highlighting this bioactive compound as a promising adjuvant in CRC immunotherapy and opening avenues for future research combining immunotherapy with PTSO in alternative models to optimize dosing and enhance translational potential.

## 1. Introduction

Colorectal cancer (CRC) remains a major global health concern, ranking as the third most commonly diagnosed malignancy and the second leading cause of cancer-related mortality worldwide [1,2]. Despite therapeutic advancements, the five-year survival rate for patients with advanced-stage CRC remains dismally low, at less than 13% [3]. This poor outcome is partly explained by the high heterogeneity of CRC, which comprises distinct molecular subtypes that influence both prognosis and therapeutic response. A clinically relevant classification is based on microsatellite status and mismatch repair (MMR) capacity, distinguishing microsatellite instability-high (MSI-H), microsatellite instability-low (MSI-L), and microsatellite stable (MSS) tumors. In particular, MSI-H or deficient MMR (dMMR) tumors present an increased neoantigen burden and generally respond well to immune checkpoint inhibitors (ICIs). By contrast, the majority of CRC cases are MSS or proficient MMR (pMMR), which show poor responsiveness to current immunotherapy approaches [4]. This disparity underscores the urgent need for strategies that remodel the tumor microenvironment and enhance immune sensitivity in resistant subtypes.

Immunotherapy, which harnesses and enhances the effect of the immune system against tumor cells, has achieved significant progress through the emergence of ICIs, chimeric antigen receptor (CAR)-T cell therapy, and experimental therapeutic vaccines, as well as the development of new strategies that modulate the tumor microenvironment to inhibit those immunosuppressive signals that prevent the immune system from attacking tumor cells [5]. However, each of these immunotherapeutic strategies faces significant limitations in CRC, mainly due to the poor responsiveness of MSS/pMMR tumors, the development of immune evasion mechanisms and the risk of off-tumor toxicity in some cases [4,6]. These therapeutic options target multiple components of the tumor microenvironment, including immune and stromal cells, as well as inhibitory signaling pathways. In this scenario, a critical mechanism of tumor escape is the expansion of cells with suppressive activity, notably regulatory T cells, myeloid-derived suppressor cells (MDSCs) and type 2 tumor-associated macrophages. These cell populations strongly hinder both innate and adaptive anti-tumor responses, thus becoming major obstacles to the efficacy of current therapies against cancer, particularly those based on immunotherapy [7]. Among these cell types, the infiltration of MDSCs into the tumor microenvironment has been linked to chronic inflammation, tumor progression, and the suppression of anti-tumor adaptive immune response. This immunosuppressive activity is mediated through the release of mediators such as arginase-1 (ARG1), reactive oxygen and nitrogen species, and inducible nitric oxide synthase (iNOS) [8,9]. Moreover, a connection between MDSCs and colon cancer stem cells (CSCs), which are a subset of tumor cells with high self-renewal and differentiation capacity that plays a central role in tumor initiation and progression, has been recently established. Indeed, MDSCs have been reported to promote CSC stemness through the release of exosomes, which reinforce CSC properties [10].

Research into new molecules capable of counteracting these suppressive mechanisms would offer great potential for the treatment of different CRC subtypes, especially those characterized by strong immune evasion. Additionally, the development of novel therapeutic strategies that enhance the recruitment of effector cells into the tumor microenvironment may also improve outcomes in tumors with deficient immune activity or low basal infiltration.

Phytochemicals, which are natural bioactive compounds produced by plants, have demonstrated a broad range of biological properties, including antioxidant, anti-inflammatory, and immunomodulatory effects. These compounds can modulate both systemic immunity and gut microbiota composition, thus offering a multifaceted approach for cancer prevention and treatment [11]. Among them, organosulfur compounds derived from *Allium* species, such as allicin, propyl-propane thiosulfonate (PTSO) and propyl-propane thiosulfinate (PTS), have shown particular promise in preclinical models of digestive disorders, including CRC [12,13,14,15]. In this scenario, *Allium*-derived compounds have been reported to reduce inflammatory mediators, reshape immune cell profiles and potentially restore anti-tumor surveillance [16,17,18]. Building on this evidence, the present study aims to evaluate the activity of PTSO, a bioactive organosulfur compound derived from *Allium cepa*, focusing on its impact on immune cell populations and its influence in tumor progression in CRC. To this end, this study employs an integrated experimental approach combining in vitro immune assays, 3D CSCs-based colonosphere models and an immune-humanized CRC mouse model to investigate how PTSO influences the tumor microenvironment and potentiate anti-tumor responses. Additionally, the study also explores the translational potential of dietary organosulfur compounds as adjuvants in CRC immunotherapy.

## 2. Materials and Methods

### 2.1. Reagents and Treatment

**Figure 1:**
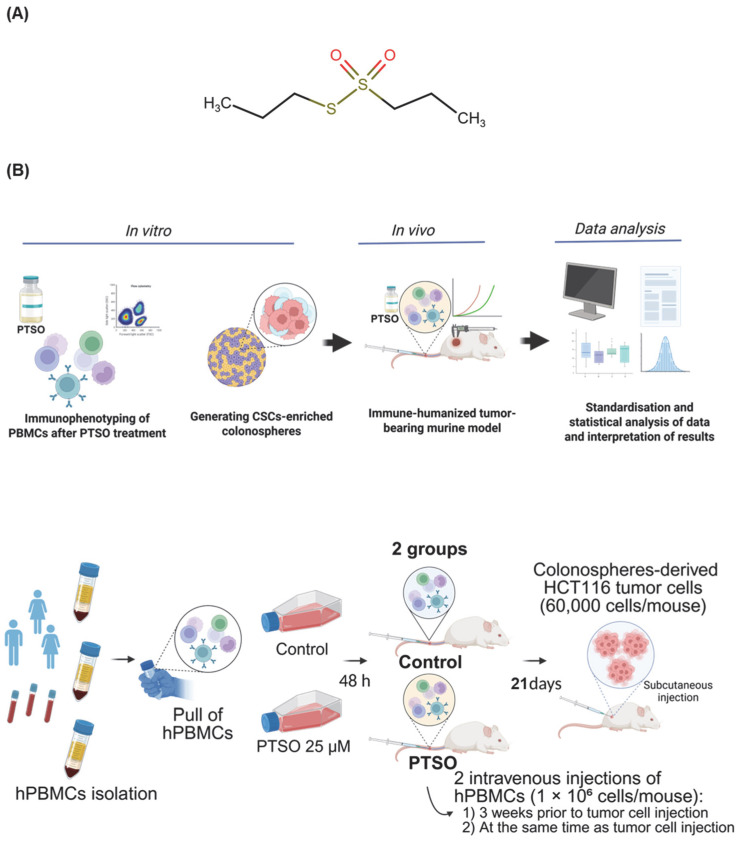
Structure of PTSO and experimental workflow. (**A**) Chemical structure of PTSO. (**B**) Top: flowchart summarizing the different experimental approaches performed in this study. Bottom: in vivo tumor xenograft model treated with PTSO-modified hPBMCs. hPBMCs were pretreated with 25 µM PTSO for 48 h and intravenously injected into mice (1 × 10^6^ cells/mouse, *n* = 9 per group). Three weeks later, 6 × 10^4^ CSC-derived colonosphere cells were subcutaneously inoculated in the right flank (1:1 Matrigel^®^/DMEM), followed by a second intravenous injection of PTSO-pretreated hPBMCs (1 × 10^6^ cells/mouse).

PTSO (Figure 1A) was obtained from *Allium cepa*, as previously reported [19], and provided by DOMCA S.A.U. at a purity of 89.6%. PTSO was initially dissolved in dimethyl sulfoxide (DMSO) and further diluted in Dulbecco’s Modified Eagle Medium (DMEM) to obtain the final working concentrations. The selection of PTSO concentrations was based on prior in vitro studies [13,20]. Unless otherwise indicated, all reagents were obtained from Sigma-Aldrich (Madrid, Spain).

### 2.2. Generating CSCs-Enriched Colonospheres

The human colorectal carcinoma cell line HCT116 was used to generate CSCs-enriched colonospheres, according to the patented protocol WO2016020572A1 [21]. Cells were obtained from the Center for Scientific Instrumentation (University of Granada, Spain) and cultured in serum-free DMEM/F-12 medium supplemented with 1× B-27 supplement minus vitamin A (Invitrogen, Waltham, MA, USA), 4 ng/mL heparin, 10 µg/mL insulin (Insulin–Transferrin–Selenium, Invitrogen), 1 µg/mL hydrocortisone, 10 ng/mL each of epidermal growth factor, fibroblast growth factor, interleukin-6, and hepatocyte growth factor (Miltenyi Biotec, Bergisch Gladbach, Germany; Auburn, CA, USA). Primary colonospheres were cultured in ultra-low attachment plates (Corning^®^ Costar^®^, Glendale, AR, USA) for 72 h. Subsequently, they were washed with PBS, disaggregated using TrypLE™ enzyme (Thermo Fisher Scientific Inc., Waltham, MA, USA) at 37 °C for 5 min, and neutralized with FBS-containing medium. After washing, single-cell suspensions were seeded in fresh ultra-low attachment plates with the same conditioned medium to generate secondary CSCs-enriched colonospheres.

### 2.3. Isolation and Culture of Human PBMCs

Human peripheral blood mononuclear cells (hPBMCs) were isolated from healthy donors following written informed consent. Cells were obtained via density gradient centrifugation using Lymphoprep™ (StemCell Technologies, Vancouver, BC, Canada) and cultured in RPMI 1640 (Gibco, Grand Island, NY, USA, Thermo Fisher Scientific, Waltham, MA, USA) supplemented with 10% fetal bovine serum (FBS), 1% L-glutamin, 1% penicillin-streptomycin, and 1% amphotericin B. hPBMCs were treated with vehicle (DMEM) or 25 μM for 48 h at 37 °C in a 5% CO_2_ incubator. Following treatment, cells were used for flow cytometry or in vivo experiments.

### 2.4. Immunophenotyping of PBMCs

PTSO- or vehicle-treated hPBMCs (*n* = 6 per group), both adherent and non-adherent, were collected and stained with viability dyes (Zombie Aqua™, BioLegend or eFluor™ 780, Invitrogen, Waltham, MA, USA) and fluorochrome-conjugated antibodies (Table S1). Labeled cells were acquired using a BD FACSymphonyTM A5 SORP (BD Biosciences, Franklin Lakes, NJ, USA) flow cytometer from the Center for Scientific Instrumentation (University of Granada, Granada, Spain) and analyzed with FlowJo software (v. 10.7.2).

### 2.5. Immune-Humanized Tumor-Bearing Murine Model

Animal procedures were conducted in accordance with the National Institutes of Health “Guide for the Care and Use of Laboratory Animals” and were approved by the Ethics Committee (Ref. No.07/12/2019/127). NOD scid gamma (NSG) mice were obtained from the Center for Scientific Instrumentation (University of Granada, Spain) and maintained under controlled environmental conditions (22 ± 1 °C, 55 ± 10% humidity, 12 h light/dark cycle) with ad libitum access to standard chow (Harlan Laboratories, Indianapolis, IN, USA) and water. Animal welfare was continuously monitored by qualified staff to ensure the well-being of the animals throughout all experimental procedures. ARRIVE guidelines were followed throughout the study. Mice (*n* = 9 per group, based on previous studies) were randomized into control (vehicle-treated hPBMCs) and PTSO (PTSO-treated hPBMCs) groups. Each animal received 1 × 10^6^ hPBMCs via tail vein injection. After three weeks, HCT116 CSCs-derived secondary colonosphere (60,000 cells/mouse) were subcutaneously inoculated in the right flank in a 1:1 mix of Matrigel^®^ (Cat.# 356231) and DMEM High Glucose without phenol red. A second intravenous injection of hPBMCs (1 × 10^6^ cells/mouse) was administered the same day (Figure 1B). Tumor growth was assessed twice weekly using a caliper and the tumor volume was calculated by the following Formula (1):

V = length^2^ × width × π/6 (1)

The tumor size was controlled to not exceed the maximum, 3 cm^3^, as defined by the ethics committee. At the end of the assay, animal Magnetic Resonance Imaging (MRI) was performed to evaluate tumor size and assess potential metastasis. The MRI scan was conducted at the Center for Scientific Instrumentation (University of Granada, Granada, Spain) using a BioSpec 70/20 USR MRI System (Bruker, Billerica, MA, USA). For this purpose, mice (*n* = 3) were anesthetized with isoflurane and scanned. Images were taken and high-intensity regions corresponding to tumor tissue were considered.

After mice were anesthetized intraperitoneally with ketamine (10 mg/kg) and xylazine (100 mg/kg) and subsequently euthanized by cervical dislocation, tumors were excised and weighed. Blood and tumor samples were taken and stored for further gene expression, flow cytometry, and histological analysis.

### 2.6. Immune Profiling of Blood and Tumor Samples

At the endpoint of the in vivo assay, tumors and blood samples were harvested for immune profiling. Blood was collected via cardiac puncture and subjected to red blood cell lysis. Tumor tissues were cut into small pieces and enzymatically digested in 5 mL of HBSS (40–50 mg tissue/mL) containing 400 U/mL DNase, 500 U/mL collagenase type IV and 0.09 U/mL dispase II (Roche Applied System, Penzberg, Germany). Digestion was incubated for 30 min at 37 °C with agitation, then washed twice with cold PBS containing 2 mM EDTA and filtered through a 70-μm nylon mesh. Single cell suspensions from blood were stained with anti-C45, anti-CD193, anti-CD14, and anti-CD64 (Table S2); and single cell suspensions from tumors were stained with anti-C45, anti-CD193, anti-CD14, anti-CD64, anti-CD3, anti-CD4, and anti-CD56 (Table S2). Labeled cells were acquired on a BD FACSymphonyTM A5 SORP (BD Biosciences) flow cytometer from the Center for Scientific Instrumentation (University of Granada, Granada, Spain) and analyzed using FlowJo software (v. 10.7.2).

### 2.7. RNA Extraction and Analysis of Gene Expression

Total RNA from tumor tissues was extracted using the Maxwell^®^ 16 total RNA Purification Kit (Promega, Madison, WI, USA) (Cat.# AS1340) with DNase treatment, according to the manufacturer’s recommendations. Then, RNA was reverse transcribed into cDNA, which was used for qPCR using an EcoTM Real-Time PCR system (Illumina, San Diego, CA, USA). Gene expression was normalized to GAPDH levels using the 2^−ΔΔCt^ method and all samples were measured in triplicate. The specific primer sequences are presented in Supplementary Table S2.

### 2.8. Histological Studies

Tumor sections were fixed in 4% PFA for 24 h and embedded in paraffin for haematoxylin and eosin (H&E) staining. For immunofluorescence analysis, antigen retrieval was performed prior to incubation with an anti-KI67 primary antibody (Abcam^®^, Abcam, Waltham, MA, USA, Cat.# AB15580 1:500 dilution in blocking buffer) overnight at 4 °C. Subsequently, sections were incubated with a goat anti-rabbit conjugated with AlexaFluor 488 (Abcam^®^, Cat.# AB170057, 1:1000 dilution in PBS).

Images were acquired from three random fields per slide using a light microscope, for H&E-stained sections, or a fluorescence microscope, for KI67 stained sections. Images were captured and analyzed with ImageJ Software (v. 1.54f), which was used to count KI67 positive nuclei and quantify the fluorescence intensity of the labeling with anti-KI67 antibody.

### 2.9. Statistical Analysis

Statistical analysis was conducted using the GraphPad Prism version 8 software (GraphPad Software, Inc., San Diego, CA, USA). All experimental animals were retained for analysis; no exclusion criteria were applied. Data are presented as mean ± SEM from at least three independent experiments or biological replicates unless otherwise specified in the figure legends. Data distribution was assessed for normality prior to statistical testing. For multiple group comparisons, one-way analysis of variance (ANOVA) followed by Tukey’s post hoc test was applied. A *p*-value < 0.05 was considered statistically significant.

## 3. Results

### 3.1. PTSO Reduces the Levels of Immunosuppressive Myeloid Populations In Vitro

**Figure 2:**
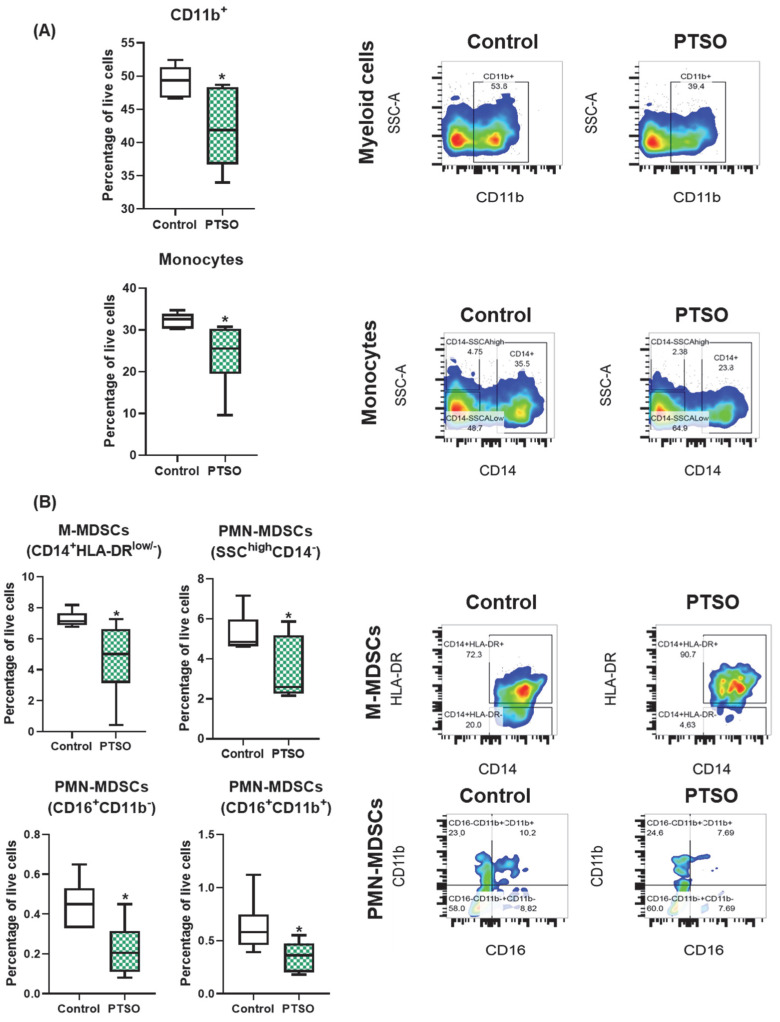
Impact of PTSO on the myeloid fraction of hPBMCs. (**A**) Effect of PTSO on the overall myeloid population (CD11b^+^) and on monocytes (CD11b^+^CD14^+^). (**B**) Effect of PTSO on myeloid-derived suppressor cell (MDSC) subpopulations. Data (*n* = 6) are presented as mean ± SEM. * *p* < 0.05 vs. Control.

To investigate the immunomodulatory potential of PTSO, we assessed its effects on myeloid subsets within hPBMCs via flow cytometry. The immunomodulatory role of this compound resulted in a significant reduction in the percentage of total CD11b^+^ myeloid cells compared to control-treated hPBMCs (Figure 2A). Notably, this decrease was predominantly driven by a marked reduction in the CD11b^+^CD14^+^ monocyte population.

Next, the effect of PTSO on MDSCs, a heterogeneous group of immunosuppressive cells broadly classified into monocytic (M-MDSC, CD14^+^HLA-DR^low/−^) and granulocytic or polymorphonuclear (PMN-MDSC, SSC^high^CD14^−^) subtypes, was also evaluated. These populations are known contributors to tumor immune evasion and progression [22]. As shown in Figure 2B, PTSO significantly reduced the proportion of both M-MDSCs and PMN-MDSCs. Within the PMN-MDSC subset, reductions were observed in both CD16^+^CD11b^+^ and CD16^+^CD11b^−^ populations. These findings indicate that PTSO can exert an immunomodulatory effect by selectively depleting immunosuppressive myeloid populations in vitro, which could impact positively on adaptive immune response.

### 3.2. PTSO Promotes Cytotoxic T Cell Expansion In Vitro

**Figure 3:**
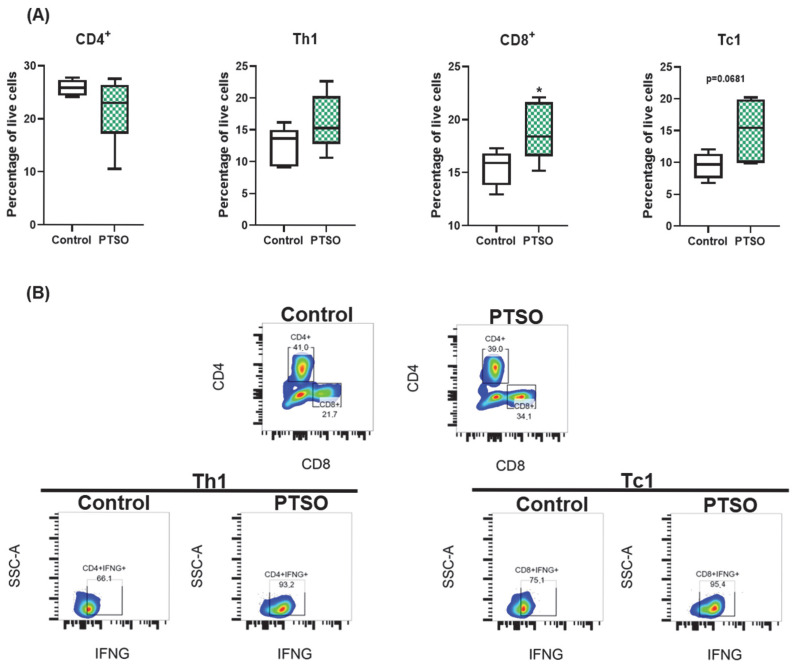
Effect of PTSO on CD4^+^ and CD8^+^ T cell subsets in hPBMCs. (**A**) Quantification of the effect of PTSO on CD4^+^, Th1, CD8^+^, and Tc1 populations in hPBMCs. (**B**) Representative gating strategy showing the effect of PTSO on CD4^+^ T cells and Th1 (CD3^+^CD4^+^IFNG^+^) and Tc1 (CD3^+^CD8^+^IFNG^+^) subpopulations. Data (*n* = 6) are presented as mean ± SEM. * *p* < 0.05, vs. Control.

The impact of PTSO on T lymphocyte subsets was also evaluated in vitro in the PBMC populations (Figure 3). As shown in Figure 3A, PTSO treatment did not significantly alter the proportion of total CD3^+^CD4^+^ helper T (Th) cells. However, there was a trend toward an increased frequency of Th1 cells (CD4^+^IFNG^+^), suggesting a shift toward a more proinflammatory phenotype. More importantly, PTSO significantly increased the frequency of cytotoxic CD3^+^CD8^+^ T (Tc) cells, accompanied by a notable rise in IFNG-producing Tc1 cells (CD8^+^IFNG^+^), indicating a skewing towards an effector anti-tumor phenotype (Figure 3A,B). These findings point to a potential role for PTSO in enhancing T cell-mediated immunity by fostering cytotoxic T lymphocyte expansion.

### 3.3. Immunomodulatory Activity of PTSO Impairs Tumor Growth in a CRC Xenograft Mouse Model

**Figure 4:**
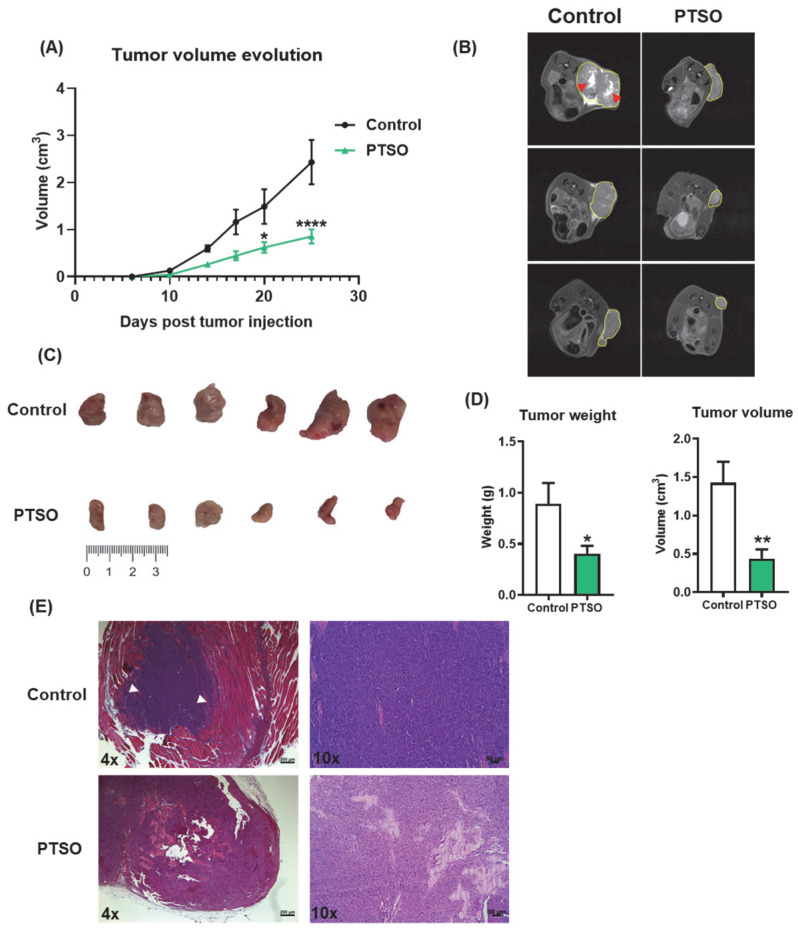
Characterization of the effect produced by the modulation of human PBMCs by PTSO in xenograft tumors. (**A**) Evolution of tumor volume (*n* = 9). (**B**) Representative cross-sectional MRI images of both groups. Tumors are outlined by yellow lines. Red arrows indicate a central region of signal enhancement, consistent with necrosis and cell death linked to heightened tumor activity (*n* = 3). (**C**) Representative images of the tumor sizes of both groups of mice. (**D**) Tumor weight and volume at endpoint (*n* = 9). (**E**) Representative histological sections of xenograft tumors stained with hematoxylin and eosin (H&E) from control and PTSO groups, scale bar: 200 μm for 4× and 50 μm for 10×. White arrows indicate areas of muscle tissue invasion by the tumor (*n* = 9). Data are presented as mean ± SEM. * *p* < 0.05, ** *p* < 0.01, **** *p* < 0.0001 vs. Control.

To translate these in vitro findings, we investigated the impact of PTSO-modulated hPBMCs on colorectal tumor development in a xenograft mouse model using HCT116 CSC-enriched colonospheres. Throughout the study, no mortality or overt signs of toxicity were observed in any of the experimental groups. Importantly, the tumors of mice that received PTSO-treated hPBMCs showed a smaller volume from day 14 post-injection, this difference being statistically significant from day 20 until the end of the assay (Figure 4A). At endpoint, MRI analysis confirmed these observations, thus revealing a marked reduction in tumor size in mice treated with PTSO-pretreated hPBMCs (Figure 4B). Conversely, the control group exhibited consistently larger volumes with more extensive tumor expansion into adjacent tissue (Figure 4B). Moreover, the greater heterogeneity in MRI signals observed in control mice indicates increased variability in tumor composition, which may be associated with hypoxia and necrotic areas (Figure 4B). Post-mortem analysis corroborated these results: mice treated with PTSO-modulated hPBMCs developed significantly lighter and smaller tumors (Figure 4C,D). Moreover, histological analysis of H&E-stained tumor sections reported notable differences between both groups (Figure 4E). In this scenario, tumors from those mice treated with control hPBMCs displayed a higher cellular density and structural integrity. Tumors from control mice exhibited dense cellularity and extensive local invasion, with muscle tissue embedded within the tumor mass (Figure 4E). Conversely, those mice administered with PTSO-pretreated hPBMCs showed prominent necrotic areas associated with loss of tumor integrity. Importantly, no evidence of adjacent tissue invasion was observed in this group (Figure 4E).

### 3.4. PTSO Modulates Tumor Cell Proliferation and Invasive Potential

**Figure 5:**
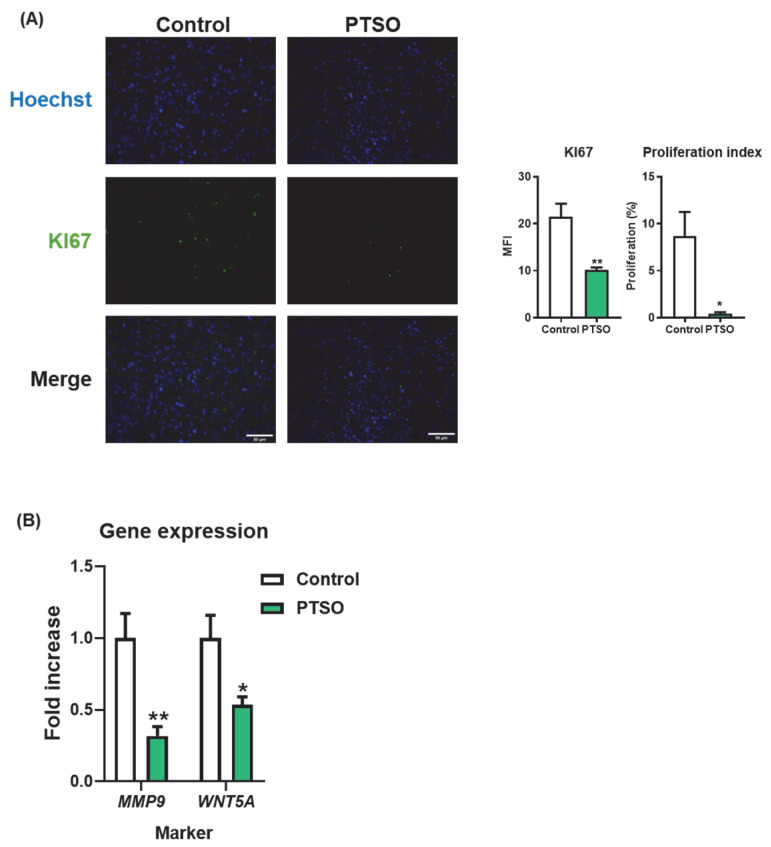
Proliferation index in xenograft tumors. (**A**) Evaluation of the proliferation process assessed by immunofluorescence using anti-KI67 staining (green); nuclei were counterstained with Hoechst (blue). Images were taken at 40× magnification; scale bar: 50 μm. (**B**) Gene expression analysis of *MMP9* and *WNT5A*. Data are presented as mean ± SEM. * *p* < 0.05, ** *p* < 0.01, vs. Control.

To evaluate whether the impact on tumor growth observed in mice receiving PTSO-pretreated hPBMCs was associated with a reduction in tumor cell aggressiveness, tumor sections were analyzed for markers of proliferation and invasion. Immunofluorescence staining of KI67 revealed a significant decrease in the number of KI67-positive nuclei in tumors from the PTSO group, indicating reduced tumor cell proliferation. This was further supported by a decrease in the mean fluorescence intensity (MFI), confirming a diminished proliferative index in these tumors. Additionally, gene expression analysis carried out in tumors from the two experimental groups revealed a significant downregulation of the proliferation and invasion marker WNT5A in mice receiving PTSO-pretreated hPBMCs (Figure 5A). Importantly, this was accompanied by reduced expression of MMP9, a matrix metalloproteinase closely associated with tumor invasion and metastasis (Figure 5B). These findings suggest that the immunomodulatory effect of PTSO on hPBMCs contributes to dampening tumor cell proliferation and invasiveness in vivo.

### 3.5. PTSO-Treated hPBMCs Enhance Tumor Immune Infiltration and Deplete the Intratumoral Myeloid Suppressor Cells

**Figure 6:**
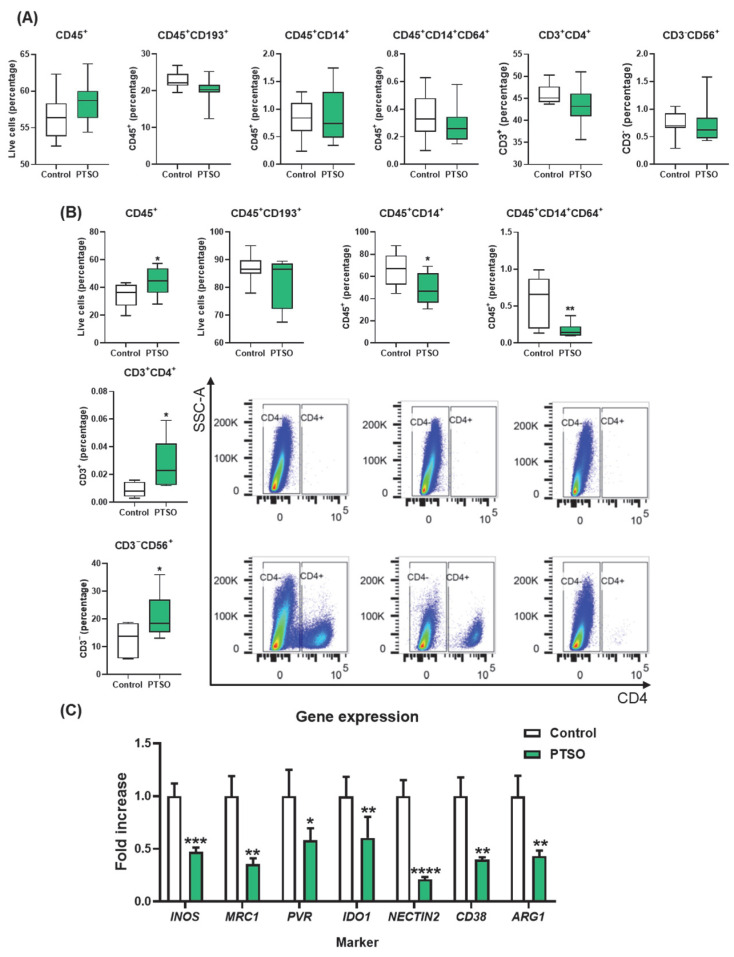
Characterization of the immunologic profile of tumor tissue of tumors xenograft modulated by human PBMCs model. (**A**) Immunologic profile of peripheral blood analyzed by flow cytometry. (**B**) Immune cell composition within tumor tissue determined by flow cytometry. (**C**) Gene expression levels of selected immune-related markers, including *INOS*, *MRC1*, *PVR*, *IDO1*, *NECTIN2*, *CD38*, and *ARG1*, assessed by qPCR. Data are presented as mean ± SEM. * *p* < 0.05, ** *p* < 0.01, *** *p* < 0.001, **** *p* < 0.0001 vs. Control.

To further dissect the immune mechanisms underlying tumor control by PTSO treatment, the presence of different immune cell populations was analyzed both in blood and in tumor tissues of xenografted mice treated with hPBMCs. Flow cytometry analysis of blood samples revealed no significant differences between both groups in the levels of total immune cells (CD45^+^), CD45^+^CD193^+^ granulocytes, neither in the general CD45^+^CD14^+^ monocytes nor particularly in those of the CD45^+^CD14^+^CD64^+^ subset (Figure 6A). Moreover, no significant changes were found in T cells (CD45^+^CD3^+^) and NK cell (CD3^−^CD56^+^) levels when compared to the two experimental groups (Figure 6A). Conversely, the analysis of immune cell infiltration into the tumor of both groups of mice revealed significant differences in the number of CD45^+^ cells, this being higher in those animals administered with PTSO-treated hPBMCs compared to Control (44.5 ± 3.13 vs. 34.2 ± 2.91), indicating an increased immune cell infiltration into the tumor (Figure 6B). Mice that received PTSO-treated hPBMCs exhibited an increased infiltration of total CD45^+^ immune cells within the tumor that was associated with a significant enrichment of CD3^+^CD4^+^ Th cells and CD3^−^CD56^+^ NK cells (Figure 6B), thus suggesting enhanced anti-tumor immune activity within the tumor microenvironment. Moreover, a significant reduction in tumor-infiltrating CD14^+^ monocytic cells was observed in the group treated with PTSO-modulated hPBMCs compared to the control group (Figure 6B). This depletion of monocytic myeloid cells in tumors was correlated with a marked downregulation in the expression of key immunosuppressive genes including MRC1, NECTIN2, and others typically associated with MDSCs such as ARG1, NOS2, IDO1, and CD38 (Figure 6C). These findings demonstrate that PTSO-pretreated hPBMCs not only enhance immune infiltration into the tumor but also reprogram the immune landscape by reducing immunosuppressive myeloid populations, ultimately fostering a more immunostimulatory tumor microenvironment.

## 4. Discussion

Immunotherapy has emerged as a transformative approach in cancer treatment, with ICIs demonstrating significant clinical benefit in various malignancies [23]. However, their efficacy is notably limited in CRC cases characterized by MSS or pMMR, where the tumor microenvironment is typically immunologically “cold” due to low immune infiltration [24]. In these contexts, combination strategies employing immunomodulators alongside ICIs are gaining interest as promising therapeutic approaches [25].

This study demonstrates, for the first time, that PTSO, a bioactive organosulfur compound derived from *Allium cepa*, exerts significant anti-tumor effects through its immunomodulatory activity. It achieves this by limiting suppressive signals and promoting the proliferation and infiltration of immune cells with anti-tumor capabilities, such as T lymphocytes and NK cells. PTSO reduced suppressive myeloid populations, particularly MDSCs, including both M-MDSCs and PMN-MDSCs subsets, which are known to hinder anti-tumor immunity. This is of particular relevance as depletion or functional reprogramming of MDSCs has been shown to enhance effector T cell infiltration and suppress tumor growth in preclinical and clinical settings [26,27,28]. These findings are consistent with a previous study published by Fujiwara, Y. et al. (2016), which demonstrated that another bioactive compound derived from *Allium cepa*, Onionin A, inhibited the immunosuppressive activity of MDSCs, thereby reducing tumor growth and lung metastasis in a subcutaneous tumor model [29].

### 4.1. Impact of PTSO on MDSCs and Their Immunosuppressive Signaling in CRC

High infiltration of MDSCs within tumors has been consistently associated with poor clinical prognosis, due to their potent suppressive effects on effector lymphocytes and NK cells [26,30]. MDSCs exert their immunosuppressive function primarily through the secretion of mediators such as ARG1 and iNOS, which inhibit effector cell activity within the tumor microenvironment. ARG1 depletes arginine, thereby restricting T cell proliferation and activation [31], while nitric oxide produced by iNOS impairs T cell function through nitration of the T-cell receptor and CD8 molecules [32]. Notably, PTSO treatment reduced the in vivo expression of these enzymes, suggesting an attenuation of MDSC-mediated immunosuppression. In CRC, MDSCs not only suppress anti-tumor immunity but also actively promote CSCs characteristics. Granulocytic MDSCs enhance CRC cell pluripotency by transferring exosomal S100A9 proteins to tumor cells. This promotes CSC formation by upregulating markers such as CD44 and CD133, especially under hypoxic conditions within the tumor microenvironment [10]. Furthermore, MDSCs in CRC are strongly influenced by IL-6 driven STAT3 marker activation. Autocrine IL-6 maintains MDSC survival through the STAT3-DNMT epigenetic axis, helping them evade necroptosis and accumulate in the tumor microenvironment [33]. Moreover, tumor cells can induce MDSC expansion in CRC, by secreting cytokines and other factors such as VEGF, iNOS, and ARG1, which reinforces immunosuppressive feedback loops.

CD38 has also been described as a pivotal marker in the MDSCs biology by contributing to their maturation. Moreover, high expression of CD38 in MDSCs has been associated with a higher immunosuppressive capacity that results in a greater ability to suppress activated T cells and promote tumor growth in comparison with CD38^low^ MDSCs [34]. Importantly, PTSO treatment resulted in a reduction in the expression of this marker in the tumors of mice that received the pretreated hPBMCs, thus contributing to the alleviation of the immunosuppressive microenvironment and the promotion of T cell activity against tumor cells. This is noteworthy because there is currently no description in the literature of any bioactive compound derived from *Allium* that has the ability to reduce the levels of this marker.

Indoleamine 2,3-dioxygenase (IDO) is an enzyme that catalyzes the degradation of tryptophan into N-formyl kynurenine and plays a critical role in mediating immunosuppression. This effect is largely attributed to the capacity of IDO to promote an environment conducive to tumor growth by recruiting and activating MDSCs, which in turn contribute to resistance against T cell-targeted immunotherapies [35]. Specifically, IDO suppresses effector T cell activation by depleting tryptophan and increasing kynurenine production, a process that facilitates the induction and expansion of regulatory T cells, further reinforcing the immunosuppressive milieu and favoring tumor progression [35]. Notably, in this study, *IDO1* expression was significantly reduced in tumors from mice treated with PTSO-pretreated hPBMCs, suggesting that this organosulfur compound may mitigate immune suppression and enhance anti-tumor immune responses, ultimately promoting tumor immunogenic cell death [25]. These results align with previous in vitro findings reporting that *Allium*-derived compounds can downregulate *IDO* expression in vitro [36].

This study also demonstrates the ability of PTSO to attenuate immunosuppressive activity by downregulating key markers such as *MRC1*, *PVR*, and *NECTIN2*. Tumor-associated macrophages are known to express the mannose receptor C-type 1 (MRC1, also referred to as CD206) on their surface, a receptor implicated in promoting tumor immunosuppression, angiogenesis, metastasis, and relapse [37]. The PTSO-induced downregulation of MRC1 may therefore enhance tumor immunogenicity, contributing to the observed anti-tumor effects in treated mice. Additionally, tumor-associated neutrophils (TANs), which are also involved in tumor progression, have been linked to the upregulation of *NECTIN2*. The expression of this marker has been reported to inhibit CD8^+^ T-cell infiltration and diminish cytotoxic T cell activity within the tumor microenvironment [38]. Consistently, administration of PTSO-pretreated hPBMCs resulted in reduced *NECTIN2* gene expression in tumors, which may have facilitated the increased infiltration of anti-tumor T cells observed in treated mice relative to controls.

The poliovirus receptor (PVR) or CD155, also plays an important role in CRC progression and immune evasion. In fact, the expression of this receptor has been found to be increased in almost 90% of CRC patients and has been strongly associated with enhanced tumor cell proliferation, migration, invasion, and metastasis. PVR facilitates immune escape through its interaction with the TIGIT receptor on T and NK cells, thereby suppressing their cytotoxic functions. These properties make PVR an attractive immunotherapeutic target in CRC [39]. In this study, mice receiving PTSO-pretreated hPBMCs exhibited significantly reduced *PVR* gene expression within the tumor, suggesting that PTSO may contribute to tumor immunogenicity by reversing immune suppression mediated by this pathway.

### 4.2. The Role of Anti-Tumor Effector Immune Cells and Their Modulation by PTSO

The immunomodulatory capacity of PTSO, demonstrated both in vitro and in vivo, was also linked to a notable increase in anti-tumor effector immune cells, particularly CD4^+^ and CD8^+^ T lymphocytes and NK cells. Importantly, characterization of CD4^+^ and CD8^+^ T cell subpopulations in vitro showed that PTSO was able to increase those producing IFNG, thus resulting in an increase in Th1 and Tc1 subsets, which are reported to be linked to a reduction in tumor progression in CRC [40,41]. This shift toward an immune profile rich in anti-tumor T lymphocytes observed in vitro is important because these cells are the main effectors in mediating tumor cell killing, and their enhanced frequency can directly translate into improved anti-tumor immunity [42]. This immunomodulatory activity was also demonstrated in vivo, since the administration of PTSO-pretreated hPBMCs led to an increase in the tumor infiltration of CD4^+^ T lymphocytes and NK cells, which was associated with a reduction in CD14^+^ monocytic cells and the downregulation of key immunosuppressive genes, as previously discussed. These findings align with the cumulative evidence suggesting that enhanced Th1, CD8^+^, and NK cell infiltration into tumors is correlated with better prognosis and improved responsiveness to immunotherapies in CRC [43,44]. Extracts derived from *Allium* sp. have also been reported to exert an immunomodulatory role that results in an anti-tumor effect derived from an increase in the infiltration of CD4^+^ and CD8^+^ lymphocytes in subcutaneous tumors induced in vivo, thus supporting the findings obtained in the present study [29,45].

The observed increase in T and NK cell tumor infiltration further suggests that PTSO may influence the expression of adhesion molecules and chemokines that guide immune cell homing to the tumor site. Immunosuppressive myeloid cells are known to impede T cell trafficking by downregulating molecules such as L-selectin; reversing these effects may help reprogram the environment to support more effective immune responses [46]. Further studies are warranted to elucidate the impact of PTSO on chemokine gradients and adhesion molecule dynamics within the tumor microenvironment.

Importantly, the enhanced immune infiltration induced by PTSO treatment was associated with a significant anti-tumor effect, as evidenced by reduced tumor volume and compromised tumor architecture in mice receiving PTSO-pretreated PBMCs. This outcome likely reflects increased immune-mediated cytotoxicity, along with impaired angiogenesis. Indeed, MDSCs are known to promote tumor vascularization by secreting pro-angiogenic factors; therefore, their depletion by PTSO may disrupt vascular support, aggravate tumor hypoxia and sensitize cancer cells to immune attack [47].

### 4.3. Cancer Stem Cells, Proliferation, and Invasion

Moreover, this anti-tumor action observed in the PTSO groups of mice was corroborated molecularly by a reduction in cancer cell proliferation markers, specifically KI67 and *WNT5A*, which is a key marker of the Wnt/β-catenin signaling pathway. Importantly, activation of the Wnt signaling pathway is a hallmark of colorectal CSCs, sustaining their self-renewal capacity, pluripotency, and tumor-initiating potential [48]. Among its components, WNT5A has been shown to modulate dependent pathways of β-catenin, promoting sphere-forming ability, and upregulating key stemness markers, including CD44, LGR5, and ALDH1 [48,49,50,51,52]. Consequently, the downregulation of *WNT5A* observed in PTSO-treated mice may not only indicate suppression of proliferative signaling, but also a direct impairment of CSC maintenance, potentially lowering the risk of tumor recurrence and therapy resistance. Additionally, these beneficial effects resulted in a reduction in the invasive capacity of tumor cells, which was manifested in the histological analysis of the control group tumor sections, with a greater infiltration of tumor cells into adjacent tissues, such as muscle tissue, compared to those mice in the group administered hPBMCs pretreated with PTSO. Moreover, this effect was correlated with a significant reduction in molecular markers involved in tumor invasion, especially *MMP9*. It has been reported that MDSCs can promote the invasive capacity of cancer cells via MMP9, thus enabling these cells to move from the primary tumor to metastatic sites [53]. In addition, single-cell sequencing experiments have reported that an increased *MMP9* expression is correlated with decreased infiltration of cytotoxic CD8^+^ T cells into the tumor, as well as increased anti-PD-1 resistance in MSI-H/dMMR CRC patients [54]. Therefore, the immunomodulatory effect of PTSO may positively impact the expression of these proliferation and invasion markers, thus resulting in the beneficial effects observed against preclinical CRC.

Taken together, our findings provide evidence for considering PTSO as a novel immunomodulatory agent capable of reducing immunosuppressive myeloid populations while boosting T and NK cell-mediated anti-tumor responses, ultimately resulting in reduced tumor burden in a CRC xenograft model. By reprogramming the tumor microenvironment from a suppressive to an immunologically active state, PTSO holds promise as a complementary agent to improve the efficacy of immunotherapies such as ICIs and adoptive T cell therapies, which are frequently limited by the presence of MDSCs and dysfunctional T cell activity.

### 4.4. Structure–Activity Relationship of PTSO

Beyond the observed immunomodulatory effects, structural considerations may provide additional insights into the potential mechanisms of action of PTSO. Although no direct studies have addressed the structure–activity relationship of PTSO, evidence from related organosulfur compounds offers valuable clues to infer its possible immunomodulatory properties. In particular, the presence of a thiosulfinate oxide moiety is likely to enable interactions with thiol-containing proteins, thereby modulating key regulators of immune signaling such as NF-κB [55,56]. In addition, the propyl substituent may influence lipophilicity, stability, and membrane permeability, thereby contributing to the bioavailability and sustained immunological activity of the compound. These features, together with the higher oxidation state of sulfur in the thiosulfinate oxide group, suggest a capacity for controlled redox reactivity and possibly H2S release, both of which are known to play roles in immune regulation [55,57]. Nevertheless, it should be acknowledged that the present study did not assess the structure–activity relationship of PTSO. As such, the specific structural determinants responsible for the observed effects, as well as the molecular targets with which PTSO may interact, remain to be elucidated. Addressing these aspects will be essential in future studies to better define the immunopharmacological properties of PTSO and to optimize its translational potential.

### 4.5. Translational Implications

Regarding translational implications, these results align with clinical data supporting the targeting of myeloid cell populations in CRC as a strategy to improve patient outcomes [58]. Patients with lower circulating MDSC levels and greater intratumoral T cell infiltration tend to respond more favorably to immunotherapy and exhibit longer survival [59]. In this context, the ability of PTSO to modulate both ends of this immune axis suggests its potential utility as an adjunct to current immunotherapeutic regimens.

### 4.6. Study Limitations and Future Directions

Certain limitations of this study should be acknowledged. The experimental data are derived from in vitro models and xenograft systems, which, although valuable, do not fully capture the complexity of human tumor–immune interactions. The heterogeneity of the tumor microenvironment in patients receiving immunotherapy involves intricate crosstalk between immune, stromal, and tumor cells that cannot be entirely replicated in preclinical models. Furthermore, while PTSO has shown promising immunomodulatory activity and its safety profile has been evaluated in vivo [60], additional studies are needed to validate its efficacy in clinically relevant settings. Future research should incorporate more advanced models, such as patient-derived organoids or ex vivo systems, to better predict clinical outcomes. Another key consideration is the phenotypic and functional heterogeneity within the myeloid compartment [61]. While this study primarily focused on major myeloid subsets, high-resolution techniques such as single-cell RNA sequencing or mass cytometry could offer deeper insights into the specific immune subpopulations modulated by PTSO. Despite these limitations, the data presented here support the development of PTSO as a promising immunotherapeutic adjuvant capable of enhancing anti-tumor immunity and reducing tumor progression in CRC.

## 5. Conclusions

The present study highlights the immunomodulatory potential of PTSO, evidenced by its ability to reduce immunosuppressive myeloid populations, activate NK cells, and promote the differentiation of T lymphocytes towards effector phenotypes. These immune changes collectively contribute to a decrease in tumor growth, reduction in cancer cell proliferation, and invasiveness. By reprogramming the tumor microenvironment from an immunosuppressive to an immunologically active state, PTSO may enhance the efficacy of current immunotherapeutic approaches. Altogether, these findings position PTSO as a promising nutritional adjuvant for CRC therapy, while future studies are warranted to validate these effects in clinically relevant models and to further investigate its impact on specific myeloid subpopulations and CSC dynamics using patients-derived systems, in order to better elucidate its translational potential.

## Data Availability

The data presented in this study are available on request from the corresponding author due to ethical and legal reasons.

## Supplementary Materials

The following supporting information can be downloaded at: https://www.mdpi.com/article/10.3390/nu17182993/s1, Table S1: Flow cytometry antibodies; Table S2: qPCR primers sequences.

## Abbreviations

The following abbreviations are used in this manuscript:

ARG1	Arginase-1
CRC	Colorectal Cancer
CSCs	Cancer stem cells
DMEM	Dulbecco’s Modified Eagle Medium
DMSO	Dimethyl sulfoxide
hPBMCs	Human peripheral blood mononuclear cells
ICIs	Immune checkpoint inhibitors (ICIs)
IFNG	Interferon gamma
iNOS	Inducible nitric oxide synthase
MDSCs	Myeloid-derived suppressor cells
MFI	Mean fluorescence intensity
M-MDSCs	Monocytic myeloid-derived suppressor cells
MRI	Magnetic Resonance Imaging
MSS	Microsatellite stability
NK	Natural Killer
NSG	NOD scid gamma
pMMR	Proficient mismatch repair
PMN-MDSCs	Polymorphonuclear Myeloid-derived suppressor cells
PTSO	Propyl-Propane Thiosulfonate
Tc	T cytotoxic
Th	T helper
