# Supplementary material for: Modulation of Human Immune Cells by Propyl-Propane Thiosulfonate (PTSO) Inhibits Colorectal Tumor Progression in a Humanized Mouse Model
Source: Nutrients. 2025 Sep 18;17(18):2993. doi: 10.3390/nu17182993 (PMC12472244; doi:10.3390/nu17182993)
Supplement: Supplementary file 1 [file nutrients-17-02993-s001.zip › nutrients-3867956-supplementary.pdf]

Table S1. Flow cytometry antibodies.

Antigen	Fluorochrome	Company	Cat. Number
CD45	APC-eFluor780	Invitrogen	47-0459-42
CD193	BV605	BD Horizon	564188
CD14	PERCP	Becton Dickinson	345786
CD64	PE	SONY	2125040
CD3	PERCP	BD Horizon	345766
CD4	PE	BioLegend	300508
CD56	APC	BioLegend	318309

Table S2. qPCR primers sequences.

Gene	Primer sequence 5′-3′	Specie	Annealing T (ºC)	RefSeq accession number
GAPDH	FW 5'-CCATCACCATCTTCCAGGAG-3'<br>RV 5'-CCTGCTTCACCACCTTCTTG-3'	mouse/ human	60	NM_001289726.1
NOS2	FW 5'- GAACATCCCAAATACGAGTGG -3'<br>RV 5'- TGGTCACATTCTGCTTCTGG -3'	human	58	NM_016368.5
MRC1	FW 5'-AGCCAACACCAGCTCCTCAAGA-3'<br>RV 5'-CAAAACGCTCGCGCATTGTCCA-3'	human	67	NM_002438.4
IDO1	FW 5'- TTGTTCTCATTTCGTGATGG -3'<br>RV 5′- TACTTTGATTGCAGAAGCAG -3'	human	55	NM_002164.6
PVR	FW 5'-GAATCCTGGTTTTTCTGATCC-3'<br>RV 5'-ATAGGAGACATGCCCATTAG-3'	human	55	NM_006505.5
NECTIN2	FW 5'-ATGATGACAACTGGTACCTC-3'<br>RV 5'-GAAGGTGGTATTGAACAAGAC-3'	human	55	NM_001042724.2
CD38	FW 5'- CAGACCTGACAAGTTTCTTC -3'<br>RV 5'- GATGACATAAACCACAAGGAG -3'	human	55	NM_001775.4
ARG1	FW 5'- ACTAGGAAGAAAGAAAAGGC -3'<br>RV 5'- TCTTCTGTGATGTAGAGACC -3'	human	55	NM_000045.4
MMP9	FW 5'- TGGGCTACGTGACCTATGAC -3'<br>RV 5'- CAAAGGTGAGAAGAGAGGGC -3'	human	58	NM_004994.3
WNT5A	FW 5'- ATTAATTCTGGCTCCACTTG -3'<br>RV 5'- GGTTATTCATACCTAGCGAC -3'	human	55	NM_003392.7
